# Determination of Selenium in Common and Selenium-Rich Rice from Different Areas in China and Assessment of Their Dietary Intake

**DOI:** 10.3390/ijerph17124596

**Published:** 2020-06-26

**Authors:** Liuquan Zhang, Yanbin Guo, Kehong Liang, Zhongqiu Hu, Xiangdong Sun, Yong Fang, Xiaohong Mei, Hongqing Yin, Xianjin Liu, Baiyi Lu

**Affiliations:** 1 National-Local Joint Engineering Laboratory of Intelligent Food Technology and Equipment, Key Laboratory for Agro-Products Nutritional Evaluation of Ministry of Agriculture and Rural Affairs, Laboratory of Quality and Safety Risk Assessment for Agro-Products Storage and Preservation of Ministry of Agriculture and Rural Affairs, Key Laboratory of Agro-Products Postharvest Handling of Ministry of Agriculture and Rural Affairs, Zhejiang Key Laboratory for Agro-Food Processing, Zhejiang International Scientific and Technological Cooperation Base of Health Food Manufacturing and Quality Control, College of Biosystems Engineering and Food Science, Zhejiang University, Hangzhou 310058, China; 11613041@zju.edu.cn; 2 Fuli Institute of Food Science, Zhejiang University, Hangzhou 310058, China; 3 Institute of Food Quality Safety and Nutrition, Jiangsu Academy of Agricultural Sciences, Nanjing 210014, China; jaasliu@jaas.ac.cn; 4 College of Resources and Environmental Sciences, China Agricultural University, Beijing 100083, China; guoyb@cau.edu.cn; 5 Institute of Food and Nutrition Development, Ministry of Agriculture and Rural Affairs, Beijing 100081, China; liangkehong@caas.cn; 6 Laboratory of Quality and Safety Risk Assessment for Agro-products, Ministry of Agriculture and Rural Affairs, College of Food Science and Engineering, Northwest A and F University, Yangling 712100, China; hzq@nwsuaf.edu.cn; 7 Laboratory of Quality and Safety Risk Assessment for Agro-Products, Ministry of Agriculture and Rural Affairs, Institute of Agro-Products Quality and Safety, Heilongjiang Academy of Agriculture Science, Harbin 150086, China; xdsun65@yahoo.com; 8 College of Food Science and Engineering, Nanjing University of Finance and Economics, Nanjing 210046, China; fangyong10@nufe.edu.cn; 9 Laboratory of Quality and Safety Risk Assessment for Agro-Products Storage and Preservation, Ministry of Agriculture and Rural Affairs, College of Food Science and Nutritional Engineering, China Agricultural University, Beijing 100083, China; mxh@cau.edu.cn; 10 Academy of Agricultural Sciences, Enshi Tujia and Miao Autonomous Prefecture, Enshi 445000, China; yhq781008@163.com

**Keywords:** ICP-MS, selenium-rich rice, selenium content, the qualified rate, dietary selenium intake, risk index

## Abstract

In this study, 41 common rice varieties and 211 selenium-rich rice varieties from ten representative areas in China were collected in 2017–2019. The selenium contents of rice were analyzed with optimized inductively coupled plasma mass spectrometry (ICP-MS). Selenium concentrations of common rice and selenium-rich rice ranges were 0.81–7.26 and 0.76–180.73 µg/100 g, respectively. The selenium contents in selenium-rich rice from different areas were significantly different (*p* < 0.001) while those in common rice from different areas were not. The selenium-rich rice in Harbin and Keshan showed the lowest selenium level and those from selenium-rich areas (Enshi and Ankang) were highest. Based on the estimation of the risk assessment software @risk7.0 (Palisade Corporation, New York, NY, USA), the consumption of selenium-rich rice can effectively increase dietary selenium intake for the population. However, the risk index of P95 (Percentile 95) selenium exposure at the tolerable upper intake level for children at 2–14 years old exceeded 100%, with potential risk currently. Therefore, the consumption of selenium-rich rice should be properly monitored for young children and adolescents.

## 1. Introduction

In 1973, the United Nations Health Organization declared that selenium is a necessary trace element for humans [1,2]. In 1988, the Chinese Nutrition Society revised dietary nutrients, listing selenium as one of the 15 daily essential dietary nutrients. The total selenium in humans was found to be about 14–21 mg and was widely distributed in all tissues except fats, and mainly existing in the form of selenium protein [3,4]. Although the content of selenium is relatively low in human tissue, it is important as iron, zinc, iodine, and other trace elements [5]. Selenium promotes growth and protects cardiovascular [6] and maintains heart muscle health [7]. It is an integral part of glutathione peroxidase (GSH-Px), which works with vitamin E to eliminate free radicals and delay aging [8,9].

According to the results of a recent nutrition survey conducted by the Chinese Nutrition Society, we found that the daily intake of selenium of Chinese adults was only 44.6 μg, far below the recommended daily intake by the Chinese Nutrition Society (60–250 μg/d) and the International Academy of Selenium (60–400 μg/d). Besides, due to the uneven distribution of selenium in the earth’s crust, many countries and areas have problems of selenium deficiency or low selenium, such as New Zealand, Finland, and parts of China [10,11]. These areas have tried to increase the selenium intake of animals and humans by planting selenium-rich crops or adding selenium in animal feed [12]. In China, there are 72% of low-selenium areas, and people in these areas could not take in enough selenium if they only consume local food. Therefore, it is of great significance to advocate selenium supplementation to improve selenium intake and disease prevention [13,14]. Since inorganic selenium is more toxic than organic Se to mammals at high doses, dietary (organic) selenium is the preferred form for dietary supplements [15]. As a result, selenium-rich foods have become an emerging functional food, especially since 2012, the consumption of selenium-rich eggs, selenium-rich rice, selenium-rich tea, selenium-rich potatoes, and other products with selenium-rich signs has become a trend. Their prices were 50–150% higher than those of the same type of common products [16].

China is the country of origin and an important producer of rice. Seventy percent of the population choose rice as a staple food [8,17]. The amount of selenium obtained from staple foods per day accounts for more than 60% of the total dietary selenium [18]. Therefore, rice has become a very important source of selenium intake for the population in China [19,20]. Due to the differences between rice varieties [21,22], soil environment [15], selenium source types [23], and selenium application methods, the selenium content of selenium-rich rice in the market varies [24], and it is difficult to accurately assess the dietary selenium intake of the population. At the same time, the current testing and certification standards system of selenium-rich rice is not ideal, and the market supervision is incomplete, resulting in shoddy selenium-rich rice, such as inorganic selenium incorporation to improve the content of selenium, causing potential health risks [25]. At present, most studies focus on the selenium content of selenium-rich rice, while there are few reports on its health hazard as a result of insufficient or excessive selenium intake [26]. Therefore, it is necessary to conduct market research, carry out in-depth research on selenium content and selenium type of selenium-rich rice, and estimate selenium intake and risk from selenium-rich rice for the population in different areas, ages, and genders.

Based on the above problems, we collected selenium-rich and common rice samples from ten representative areas (seven common areas, two selenium-rich areas, and one selenium-deficient area) in 2017–2019, focusing on the following issues [27]: (1) to define the background value of selenium content of common rice in China; (2) to detect selenium content of selenium-rich rice from different areas in China; (3) to analyze the proportion of organic selenium and inorganic selenium in selenium-rich rice and assess its health benefits; (4) to evaluate the dietary selenium intake and risk for population in different areas, ages, and genders.

## 2. Materials and Methods

### 2.1. Selection of Sampling Sites and Sampling

In this study, seven representative common areas were selected according to the geographical distribution from north to south in China: Northeast (Harbin, Heilongjiang Province), North China (Beijing), East China (Hangzhou, Zhejiang Province), Central China (Wuhan, Hubei Province), Southwest (Chongqing), Northwest (Xi’an, Shaanxi Province), South China (Guangzhou, Guangdong Province) [28]. Meanwhile, Ankang, Shaanxi Province, and Enshi Autonomous Prefecture of Hubei Province were chosen as typical selenium-rich areas; Keshan County, Heilongjiang Province was selected as a typical selenium-deficient area (Figure S1 in Supplementary Material) [28]. Three sampling points were needed in each area. At least five common rice samples and five selenium-rich rice samples were collected at each sampling point. A single sample was not less than 5 kg. A mixed sample was made by mixing five common rice samples at each sampling point. A total of 41 non-selenium-enriched rice samples and 211 selenium-enriched rice samples were collected in this study.

### 2.2. Sample Pretreatment for Total Selenium Detection

Referring to the National Standards of China, GB5009.93-2017 “Food Safety National Standard - The Determination of Selenium in Food”, inductively coupling plasma mass spectrometry (ICP-MS) method were used. The crushed rice sample (0.5 g) was placed into the digestive tube, and 7 mL nitric acid (analytic grade, China National Pharmaceutical Group Co., Ltd., Beijing, China) was added. It was pre-dissolved for 30 min in graphite furnace (TOPEX, Preekem, Shanghai, China) at 110 °C in the ventilation cabinet. Then it was cooled to room temperature, and dissociated by putting the digestive tube into the casing following the microwave digestion procedure (0–3 min: 120 °C; 4–6 min: 150 °C; 7–9 min: 180 °C; 10–30 min: 200 °C). The digestive tube was cooled to room temperature, moved into a graphite furnace at 110 °C and concentrated to 0.5–1.0 mL. Then it was transferred to a 50 mL capacity bottle and distilled with distilled water. After centrifugation (Eppendorf, Hamburg, Germany) at 10,000 r/min for 10 min, the supernatant was passed through a 0.22 μm aqueous filter to be tested. At the same time, a blank sample was prepared without the addition of crushed rice sample. 

### 2.3. Sample Pretreatment for Inorganic Selenium Detection

The crushed rice (1.0 g) was placed into a 50 mL centrifuge tube, and 10 mL 2% nitric acid was added in it. After vibration in a 60 °C water bath (Jiangdong, Suzhou, China) for 2 h, it was cooled to room temperature. Then it was transferred into a 25 mL volumetric flask and brought to volume by 2% nitric acid. After centrifugation at 10,000 r/min for 10 min, the supernatant was passed through a 0.22 μm aqueous filter to be tested. At the same time, a blank sample was prepared.

### 2.4. Detection of Organic Selenium

Total selenium minus inorganic selenium, that is, the content of organic selenium.

### 2.5. Inductively Coupled Plasma Mass Spectrometry (ICP-MS) Working Conditions

The instrument was adjusted to the working condition with a standard tuning solution. The internal standard solution which might be a standard solution or a sample solution was introduced into the atomization chamber by an ICP-MS (NexION 300X, PerkinElmer, Waltham, MA, USA) peristaltic pump. After atomization, it was ionized in a high-temperature plasma. Finally, it was detected by a mass analyzer and quantified by the standard curve. ICP-MS working conditions were shown in Table S1 in the Supplementary Material.

### 2.6. Quality Assurance and Quality Control Data

Firstly, in the weighing process of the samples, the samples were mixed uniformly. Parallel samples were taken for digestion and determination, and at least one pair of parallel determinations were made for each sample. Secondly, they were measured with ICP-MS. After the system startup, the tuned liquid was used to calibrate the instrument so that it met the requirement in all parameters including ICP-MS atomizing gas flow rate, rectangular tube, ion lens, oxide, double charge, and resolution. For each batch of sample analysis, at least two reagent blanks and standard reference materials should be tested to ensure the reliability of the test results. Thirdly, when each batch of samples was tested, the blank method was used to confirm that the reagent and utensils used did not contain the measured elements or the content of the measured elements was far lower than that in samples. When the method blank was detected to contain the interference of the measured elements, this batch of samples should be treated again. Finally, during the sample testing process, the standard solution was injected every 20 samples to ensure the stability of the instrument. When the recovery of selenium measured in the standard solution exceeded the range of 90–110%, the standard curve was recalibrated.

### 2.7. Precision and Stability of the Detection Method

Under the optimized test conditions, a 2% nitric acid blank solution was measured 11 times in parallel, and the standard deviation of the selenium element was calculated. By deviation determination method at three times of the standard, the detection limit was 0.001 mg/kg, and at ten times of the standard deviation determination method, the determination limit was 0.004 mg/kg. The quantitative lower limit of selenium was lower than the measurement standard of multi-element in food (GB 5009.268-2016) (detection limit and determination limit requirements were 0.001 mg/kg and 0.004 mg/kg, respectively), following the standard method requirements.

The standard solution of selenium (1000 μg/mL, National Nonferrous Metals and Electronic Materials Analysis and Test Inge., Beijing, China) with a concentration of 0.05 μg/L, 0.1 μg/L, 0.5 μg/L, 1 μg/L, 5 μg/L, was determined under optimized experimental conditions, and the standard curve was drawn with a mass concentration as horizontal coordinate, signal strength as the vertical coordinate and the regression equation was: *y* = 603.90 *× x* + 10.29. With a correlation coefficient of 1.0, it is satisfactory to meet the detection requirements.

According to the sample treatment method of total selenium, the total selenium content of citrus leaf standard sample GBW10020 (Geophysical Geochemical Survey Research Institute, Beijing, China) was measured eight times under the optimized ICP-MS working conditions, with an average value of 0.199 mg/kg, and a relative standard deviation of 3.39%.

The recovery experiment (Table 1) was carried out according to the total selenium sample treatment method, the total selenium recovery rate ranged from 98.17% to 99.98%, the average value was 99.11%, and the relative standard deviation was between 0.50% and 4.15%. The precision requirement of GB 5009.268-2016 for total selenium is: when less than or equal to 1.0 mg/kg and greater than 0.1 mg/kg, the absolute difference between the two independent measurements obtained under repetitive conditions shall not exceed 15% of the arithmetic mean value. Since the relative standard deviation of the total selenium recovery experiment in this experiment was 4.15%, the requirement of the standard method was satisfied.

According to the inorganic selenium sample treatment method, the recovery experiments were carried out (Table 1), the inorganic selenium recovery rate was between 83.19% and 98.93%, the average value was 92.41%, the relative standard deviation was between 0.02% and 7.22%. Those reported previously in the literature had specified that inorganic selenium content should be in the range of 0.01 mg/kg to 0.10 mg/kg, the absolute difference value of the two independent test results obtained in the repetitive conditions shall not exceed 40% of the arithmetic average, and the deviation from the standard of the inorganic recovery experiment in this research was 7.22%, that the requirements of the standard method were satisfied.

### 2.8. Assessment Model of Daily Dietary Selenium Intake

In this research the risk assessment software @risk 7.0 based on Monte Carlo simulation technology was used to quickly assess dietary selenium intake of different population groups. That was, a random sampling of Monte Carlo simulations has proceeded within a possible range of values, and the probability distribution of all values was used to simulate population intake, to estimate the amount of dietary selenium intake from common or selenium-rich rice in various areas, ages and genders groups in China. Based on the measured selenium content in common and selenium-rich rice and the average rice consumption for different population groups, the calculation method of individual intake was shown in formula [27]:

(1) $$EDI=\frac{C\times PIR\times ED\times EF}{AT}$$

EDI is the estimated daily dietary selenium intake for different populations (μg/d); C is selenium content (mg/kg) in common or selenium-rich rice samples; EF is exposure frequency (360 ds/year), ED is exposure period (years), and AT is the average exposure time (equivalent to EF × ED); PIR is the daily rice intake for different populations (g/d). The PIR data in this study was derived from *The Study of Nutrition and Health of Chinese Residents III-2002* (Table S2 in Supplementary Material showed daily rice intake of different age and genders groups; Daily rice intake of the population in different areas: Hangzhou-303.25 g/d, Beijing-133.33 g/d, Wuhan-243.15 g/d, Guangzhou-252.10 g/d, Xi’an-110.30 g/d, Chongqing-293.93 g/d, Harbin-149.00 g/d, Enshi-307.45 g/d, Ankang-64.99 g/d, Keshan-149.00 g/d).

### 2.9. Risk index Calculation

Risk index R was calculated by the formula (2) [29]. The smaller the R-value, the lower the risk. The risk was acceptable when R was less than 100%. An unacceptable risk was indicated when R was over 100%.

(2) $$R=\frac{EDI\times 100}{\left(S-EDI’\right)}$$

EDI was dietary selenium intake from rice per day for different populations groups (mg/kg); S was evaluation criteria (μg/d), referring to the recommended intake dose (RDI) of 60 μg/d or tolerable upper intake level (UL) of 400 μg/d; EDI’ was selenium intake from other dietary components.

### 2.10. Statistical Analysis

Selenium levels in selected rice samples were presented as the mean values of at least 6 independent measurements of a sample. Data analyses were performed using SPSS 20.0 (SPSS Inc., Chicago, IL, USA) and Origin 9.1 (OriginLab, Northampton, MA, USA).

## 3. Results and Discussion

### 3.1. Selenium Content of Common/Selenium-Rich Rice Samples from Different Areas in China

**Figure 1:**
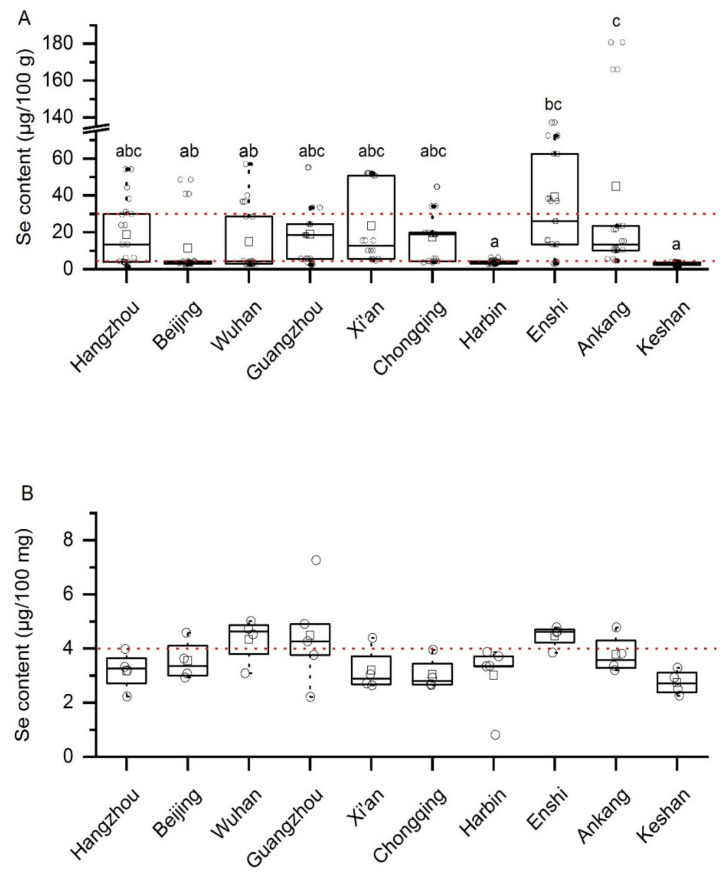
Box diagram of total selenium content in selenium-rich and common rice in 10 sampling areas. (**A**) Box diagram of selenium content in selenium-rich rice: the two red lines are 4 μg/100 g and 30 μg/100 g specified in the national standard of the People’s Republic of China (GB/T 22499–2008) of selenium-rich rice respectively. “a, b, c” represents the result of pairwise comparison of Tukey HSD, and the same letter indicates no significant difference between the two groups; (**B**) Box diagram of selenium content in common rice: the red line is the lower limit of the standard GB/T 22499–2008.

In general, the selenium contents of the selenium-rich rice were mostly in the range of 5–30 µg/100 g (with Xi’an and Enshi 5–60 µg/100 g) (Figure 1A), while those of the common rice was mostly in the range of 2.5–5 µg/100 g (Figure 1B). These results suggested that none of the common rice was grown on soils with high plant-available selenium in any of the areas sampled. If they had been, the selenium contents of these rice would be well above 5 µg/100g, even reach 30 µg/100 g. Selenium in the soil varies greatly even with small distances, i.e., even high-selenium areas have a mixture of high-selenium and low-selenium soils. Hence, we speculate that those selenium-rich rice with selenium contents lower than 10 µg/100g in notable high selenium areas such as Enshi and Ankang may not be grown on high selenium soils in these areas. 

It was shown through a one-way analysis of variance (one-way ANOVA) that selenium content values varied significantly in the selenium-rich rice samples from 10 areas (*p* < 0.001). The Tukey HSD (Honestly Significant Difference) method was used to compare in pairs, with the results shown in Figure 1. The average selenium content of selenium-rich rice from Harbin and Keshan was only 3.90 µg/100 g and 2.90 µg/100 g, respectively. The mean values selenium content of selenium-rich rice samples in Hangzhou, Beijing, Wuhan, Guangzhou, Xi’an, and Chongqing were within the standard range (4–30 µg/100 g) of selenium-rich rice (Figure 1A), but they were not significantly different from selenium-rich rice in Harbin and Keshan. The reason might be that selenium-rich rice sampled in the same area varied greatly in its selenium content. For example, the highest selenium content of selenium-rich rice in Hangzhou was 54.24 µg/100 g, exceeded the upper limit of the selenium-rich rice standard, while the lowest was only 3.89 µg/100 g, below the lower limit of the selenium-rich rice standard. Enshi and Ankang are selenium-rich areas where samples would be locally produced, so their selenium content was high, and even their average value was higher than the upper limit of selenium-rich rice standard. For example, the selenium content of Enshi and Ankang’s selenium-rich rice could have up to 137.24 and 180.73 µg/100 g, respectively. The first quartile (First Quartile, Q1) and the second quartile (Second Quartile, Q2) of selenium content of Enshi selenium-rich rice and the Q1, Q2, and Q3 values of selenium content in Ankang samples were within the standard range, indicating that most of the samples in both areas meet the standard. The large difference in the selenium content of selenium-rich rice might also be related to processing degree, the study found that with the improvement of processing degree, the selenium content of rice decreased [30], which resulted in the selenium content of selenium-rich rice might not be able to meet the standard value [31].

There was no significant difference in the selenium content of the common rice from ten areas (*p* = 0.094). Samples in some areas had selenium content higher than the lower standard limit for selenium-rich rice, such as some rice samples in Wuhan, Guangzhou, and Enshi (Figure 1B). It showed that the selenium content of common rice in China was relatively low, which was consistent with the conclusion that 72% of China’s land area was deficient in selenium, as other studies.

In this study, the mean selenium contents of common and selenium-rich rice were 3.59 µg/100 g and 19.53 µg/ 100 g, respectively. The concentration in this research was compared with the data in those reported previously (Table 2). The comparison showed that the rice produced in the United States [32], Egypt [33], and India [34] had a higher selenium content, while rice in the major producing and consuming countries such as China [35], Japan [36], and other countries had lower selenium content. This phenomenon is usually caused by the selenium content in the soil, which had directly affected the selenium content of rice in different countries. In our study, the selenium concentration range of common rice was 0.81–7.26 µg/ 100 g, which was consistent with the data by Chen in 2002 [35]. The selenium content of selenium-rich rice was much higher than that in common rice, indicating that the selenium-rich rice on the market have higher selenium content than common rice, and the existing selenium-rich rice production technology is effective for the production of selenium-rich rice, thereby increase selenium intake for the population. Meanwhile, according to the research results of Panigati et al. in 2007 [37], it could be found that the selenium concentration in red rice and black rice was higher than that in white rice. Besides, in studies that have been frequently reported, it has also been proved that the content of anthocyanins and phenols in black rice and red rice was higher than that of white rice [38]. Therefore, red rice and black rice foods could be considered as a better source of natural nutrients. During the selenium enrichment process of white rice, most of the selenium accumulated in the rice husk [37].

### 3.2. Distribution of Inorganic and Organic Selenium in Selenium-Rich Rice Samples

The production of selenium-rich rice was the process of selenium element in selenium-rich soil or selenium fertilizer applied during rice growth entering the internal circulatory system of plants. By biotransformation, it was involved in the synthesis of proteins, and the major form of selenium in rice was organic selenium. This is known as agronomic biofortification. Because of the high nutritional value and the high price of selenium-rich rice, “selenium-rich rice” sold in the market was possible to be artificially added with inorganic selenium to increase selenium content. This is process fortification. To investigate the distribution of inorganic and organic selenium in selenium-rich rice in this research, twenty batches of representative rice (total selenium content: 4.053–18.956 μg/100g) were selected according to the total selenium test results. The results (Table S1 in Supplementary Material) showed that the proportion of organic selenium in the samples examined was 62.34–89.54%. Overall, inorganic selenium accounts for 10–38%, and that of the inorganic selenium was 10.46–37.66%. If some of these rice samples were grown on high selenium soil, some of the selenium in the grain could be expected to be in the form of the selenate, which may be the cause for the relatively high proportion of inorganic selenium in the sampled rice.

### 3.3. Dietary Selenium Intake and Risk Index of Common/Selenium-Rich Rice in Different Areas

**Figure 2:**
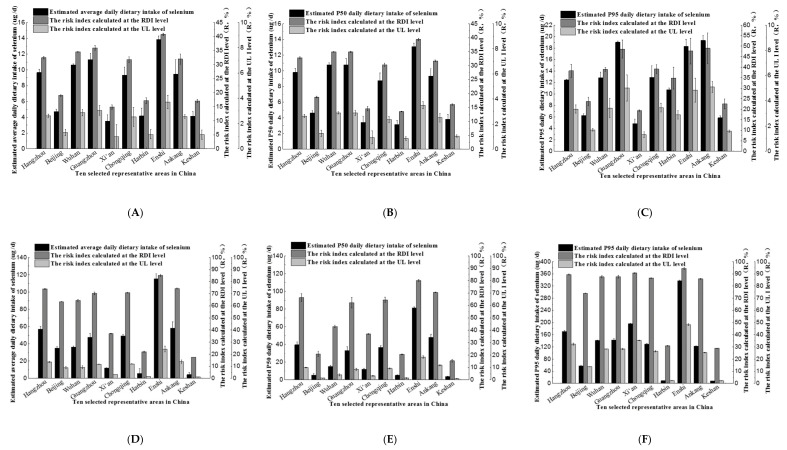
Dietary selenium intake and risk assessment of the population in different areas when they only eat common rice (**A)**–(**C**) or selenium-rich rice (**D)**–(**F**) (calculated by a standard person weighting 70 kg). When the risk index was calculated, the actual daily dietary selenium intake of the standard person was designated as 39.90 μg/d, which was derived from the data of the “national dietary nutrition survey” of the Chinese nutrition society in 2012. The recommended intake dose (RDI) (recommended daily intake) was designated as 60 μg/d and UL (tolerable upper intake level) as 400 μg/d stipulated by the Chinese nutrition society.

Using the Monte-Carlo simulation formula [27] to assess the selenium intake of common/selenium-rich rice for the population (Using the parameters of a standard Chinese) from ten areas. The average exposure, P50 (Percentile 50) exposure, and P95 exposure of the population in each area were given. At the same time, the risk index R was calculated by the formula (2). The results showed (Figure 2A–C) that the average intake of selenium for the population through common rice was generally low, which ranged from 3.52 to 13.84 μg/d, and the P95 exposure was 4.83–19.32 μg/d. With the recommended daily dietary selenium intake of 60 μg/d as the evaluation criteria, the intake risk of selenium from common rice products was uniformly very low, where the risk index R was less than 100%. Even for the P95 exposure, the risk index of different population groups in all areas was no more than P50, with little risk on human health.

Additionally, Figure 2D–F showed that the average selenium intake for the population from selenium-rich rice in ten areas was between 4.25–116.95 μg/d, and P95 exposure was 9.38–341.29 μg/d. With the recommended daily dietary selenium intake of 60 μg/d as the evaluation criterion, the risk index of P95 exposure was higher, which was close to 100%. While by taking the upper limit of selenium tolerance UL (Tolerable Upper Intake Levels) as the evaluation index, the risk of selenium intake from selenium-rich rice products was uniformly very low, the risk index was much lower than 100%, and the risk index of P95 exposure did not exceed 60%, which indicated that selenium-rich rice selected in this study was relatively safe and did not have the risk of excessive selenium intake.

### 3.4. Dietary Selenium Intake and Risk Index of Common Rice Consumed by Different Age and Genders Groups

**Figure 3:**
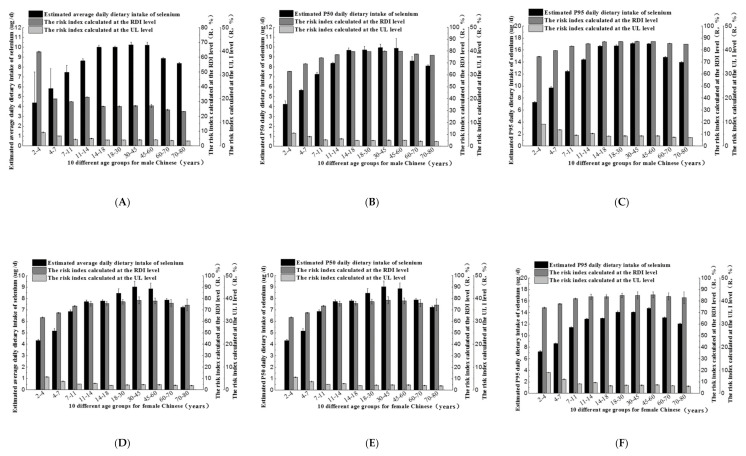
Dietary selenium intake and risk assessment of male (**A**–**C**) and female (**D**–**F**) population in different age groups when they only eat common rice. When the risk index was calculated, the actual daily dietary selenium intake data of male and female population in different age groups was from the study by Nie JY in 2015 [29] and “dietary guidelines for Chinese residents in 2016”. The RDI and UL data of male and female population in different age groups were selected by reference to selenium intake recommendations from the FAO (Food and Agriculture Organization of the United Nations)/WHO (World Health Organization) and the NNFA (US National Nutritional Foods Association).

The Chinese population was divided into 20 different gender and age groups. The average exposure, the P50 exposure, and the P95 exposure as well as the risk indexes for dietary selenium of 20 different population groups were shown in Figure 3. With the increase of age in a population group, selenium intake increased firstly and then decreased, and rice intake was related to selenium intake of all ages. Figure 3A-C showed that in the male population groups, average selenium exposure was 4.39–10.25 μg/d, the P50 exposure was 4.25–9.93 μg/d, and the high-end exposure (P95) was 7.31–17.02 μg/d. In the female population groups (Figure 3D–F), average selenium exposure was 4.33–9.03 μg/d, corresponding to a daily P50 exposure and P95 exposure of 4.20–8.75 μg/d and 7.22–14.73 μg/d, respectively. Overall, the levels of selenium intake of males of all age groups was higher than that of female groups, showing differences.

As can be seen from Figure 3, the selenium intake of all groups was much less than the recommended lower limit of 60 μg/d, with RDI as the evaluation criterion, the risk index of the P95 exposure was higher, which was close to 90%. While with UL as an evaluation index, the risk of selenium intake from common rice in all population groups was uniformly very low, where the risk index was well below 100%. The risk index for P95 exposure was no more than 10%, so there was no risk of excessive selenium intake.

### 3.5. Dietary Selenium Intake and Risk Index of Selenium-Rich Rice Consumed by the Population in Different Ages and Genders

**Figure 4:**
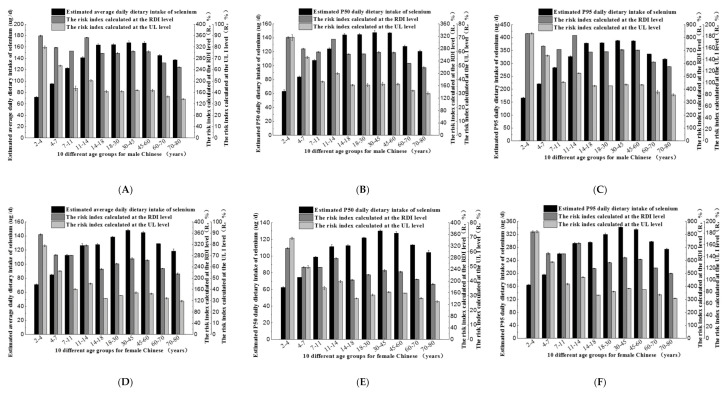
Dietary selenium intake and risk assessment of male (**A**–**C**) and female (**D**–**F**) population in different age groups when they only eat selenium-rich rice. When the risk index was calculated, the actual daily dietary selenium intake data of male and female population in different age groups was from the study by Nie J Y in 2015 and “dietary guidelines for Chinese residents in 2016”. The RDI and UL data of male and female population in different age groups was selected by reference to selenium intake recommendations from the FAO (Food and Agriculture Organization of the United Nations)/WHO (World Health Organization) and the NNFA (US National Nutritional Foods Association).

Monte-Carlo simulations were used to assess selenium intake from selenium-rich rice for 20 population groups, and to calculate their average exposure, P50 exposure, and P95 exposure and risk index, with the results shown in Figure 4. The overall trend was the same as in Figure 3. Figure 4A–C showed that average selenium exposure in the male population was from 71.86 to 167.72 μg/d, P50 exposure was from 63.23 to 147.61 μg/d, high-end exposure (P95) was from 166.16 to 387.78 μg/d. In the female groups (Figure 4D–F), average selenium exposure was from 70.94 to 147.79 μg/d, with corresponding daily P50 exposure and P95 exposure of 62.44–130.06 μg/d and 164.03–341.69 μg/d respectively, which was the same as in Figure 3. Male groups had a higher intake than women groups. Selenium intake by selenium-rich rice consumption for the population was high, so it was feasible to improve selenium deficiency for the population by consuming selenium-rich rice.

As can be seen from Figure 4, at the RDI level, the risk index for all population groups was higher than 100%, the risk index was highest for the 2–4 and 11–14 years old, and the P95 risk index can be as high as 830.80% (male), 820.15% (male), 816.18% (female), and 730.83% (female), respectively (Figure 4C, F). It indicated that at high exposure levels, there was a certain risk of excessive intake from selenium-rich rice. It did not exceed the upper tolerance standard of selenium, thus selenium-rich rice selected in this study was relatively safe for all stages of the population. With UL, the upper tolerance standard of selenium as the index for evaluation, the risk of selenium intake from selenium-rich rice products remained high, with a risk index of more than 100% of P95 exposure in age groups of 2–4 years old, 4–7 years old, 7–11 years old, and 11–14 years old (Figure 4C, F), up to 184.62%. which indicated that at high exposure levels, the risk of possible excessive intake of selenium-rich rice in these age groups should be highlighted and should be properly monitored.

## 4. Conclusions

Selenium concentration ranges of common rice and selenium-rich rice were 0.81–7.26 µg/100 g and 0.76–180.73 µg/100 g, respectively. The difference in the selenium content of selenium-rich rice from ten areas varied significantly (*p* < 0.001) while those of common rice did not. The average selenium contents of selenium-rich rice of Harbin and Keshan were lower than in other areas. Tukey HSD methodology analysis results showed that the mean selenium contents of selenium-rich rice samples in Enshi and Ankang were highest. According to the composition of organic selenium and inorganic selenium in rice, inorganic selenium accounted for a small proportion. Regardless of the rice consumed was selenium-rich or not, the risk index under the average exposure, the P50 exposure, and the P95 exposure for a standard Chinese in various areas was less than 100%, indicating that health hazards were small. For different gender and age populations, in the common rice dietary model, the overall risk index was well below 100%, so there was no risk of excessive selenium intake; in the selenium-rich rice dietary model, the risk indexes of the P95 exposure at UL level for 2–14 years old were exceeded 100%. It was suggested that at high intake levels, consumption of selenium-rich rice should be properly monitored for young children and adolescents.

## Figures and Tables

**Table 1:** Recovery and relative standard deviation of total selenium and inorganic selenium.

Testing Index	Background Value of the Sample Being Tested (mg/kg)	Amount of Reference Substance Added to 0.2 g Sample (μg)	Average Recovery (%)	Relative Standard Deviation (%)
Total selenium	0.126	0.020	98.17	4.15
		0.040	99.98	3.74
		0.060	99.17	0.50
Inorganic selenium	0.057	0.024	95.11	7.22
		0.030	98.93	0.02
		0.036	83.19	4.67

**Table 2:** Selenium concentration in rice samples reported by other authors (µg/100 g, wet weight).

Food Samples	*n*	Mean	Relative Standard Deviation	Range	References
Rice	——	70.25	7.5	62.70–87.00	USA [32]
	4	1.83	9.0	2.01 ± 0.18 (white rice)	Italy [37]
	5	4.52	4.7	5.30 ± 0.10 (red rice)	Italy [37]
	3	2.05	1.9	2.67 ±0.13 (black rice)	Italy [37]
	4	3.41	9.0	4.53 ± 0.41 (white rice hull)	Italy [37]
	27	1.80	——	1.20–2.40	Spain [31]
	69	0.35	8.5	0.05–0.12	Iran [33]
	58	——	——	1.40–3.60	China [35]
	24	1.85	5.6	1.77–2.05	Greece ^f^
	69	——	——	1.60–7.00	Japan [36]
	——	4.58	5.7	0.50–9.50	India [34]
	15	6.50	4.8	1.50–13.00	Bangladesh [30]
	——	——	——	1.30–9.90	Ireland [32]
	16	19.30	5.9	——	Egypt [33]

## Supplementary Materials

The following are available online at https://www.mdpi.com/1660-4601/17/12/4596/s1, Figure S1: Map showing the geographical location and population of ten sampling areas in China where common rice and selenium-rich rice were collected. (Population data came from China’s sixth census), Table S1: Working parameters of inductively coupled plasma mass spectrometry (ICP-MS), Table S2: The daily rice intake of residents of different gender and age population groups in China, Table S3: Distribution of organic selenium and inorganic selenium in selenium-rich rice (µg/100 g, wet weight). All data and material generated or used during the study are fully available without restriction. Supplementary data associated with this article can be found in the online version.
