# Supplementary material for: Determination of Selenium in Common and Selenium-Rich Rice from Different Areas in China and Assessment of Their Dietary Intake
Source: Int J Environ Res Public Health. 2020 Jun 26;17(12):4596. doi: 10.3390/ijerph17124596 (PMC7344401; doi:10.3390/ijerph17124596)
Supplement: Supplementary file 1 [file ijerph-17-04596-s001.pdf]

**Table S1.** Working parameters of inductively coupled plasma mass spectrometry (ICP-MS).

| Instrument Parameter | Setting Value | Instrument Parameter | Setting Value |
|---|---|---|---|
| RF power | 1100 W | Analog voltage | −1862 V |
| Atomized gas flow | 0.89 L/min | Impulse voltage | 1310 V |
| Plasma gas flow | 16.0 L/min | Scanning mode | Peaking |
| Measurement model | DRC mode | Scan times | 20 times |
| $CH_4$ flow rate | 0.8 mL/min | Reading | 1 time |
| Rejection parameter q （RPq） | 0.7 | Times of repetition | 3 times |
| Lens voltage | 6.8 V | Detected signal | $^{80}Se$ |

**Table S2.** The daily rice intake of residents of different gender and age population groups in China.

| Age (Years) | Daily Intake (g/Day) | |
|---|---|---|
| | Male | Female |
| 2–4 | 116.8 | 115.3 |
| 4–7 | 154.9 | 137.5 |
| 7–11 | 199.0 | 182.8 |
| 11–14 | 229.5 | 205.5 |
| 14–18 | 266.1 | 207.5 |
| 18–30 | 266.9 | 224.9 |
| 30–45 | 272.6 | 240.2 |
| 45–60 | 271.5 | 235.2 |
| 60–70 | 236.2 | 209.4 |
| 70–80 | 222.7 | 192.7 |

**Table S3.** Distribution of organic selenium and inorganic selenium in selenium-rich rice (μg/100 g, wet weight).

| Number | Total Selenium (μg/100 g) | Organic Selenium (μg/100 g) | The Proportion of Inorganic Selenium (%) | Inorganic Selenium (μg/100 gfw) | The Proportion of Inorganic Selenium (%) |
|---|---|---|---|---|---|
| 1 | 4.053 | 2.996 | 73.93 | 1.057 | 26.07 |
| 2 | 4.350 | 3.017 | 69.36 | 1.333 | 30.64 |
| 3 | 13.385 | 10.487 | 78.35 | 2.898 | 21.65 |
| 4 | 6.180 | 4.031 | 65.23 | 2.149 | 34.77 |
| 5 | 5.610 | 4.732 | 84.35 | 0.878 | 15.65 |
| 6 | 18.455 | 12.821 | 69.47 | 5.634 | 30.53 |
| 7 | 10.102 | 8.810 | 87.21 | 1.292 | 12.79 |
| 8 | 15.446 | 12.546 | 81.23 | 2.900 | 18.78 |
| 9 | 4.613 | 3.941 | 85.43 | 0.672 | 14.57 |
| 10 | 5.517 | 4.152 | 75.26 | 1.365 | 24.74 |
| 11 | 4.349 | 2.836 | 65.21 | 1.513 | 34.79 |
| 12 | 5.907 | 3.682 | 62.34 | 2.225 | 37.66 |
| 13 | 18.956 | 16.973 | 89.54 | 1.983 | 10.46 |
| 14 | 4.352 | 3.797 | 87.24 | 0.555 | 12.76 |
| 15 | 6.350 | 5.568 | 87.68 | 0.782 | 12.32 |
| 16 | 13.480 | 11.681 | 86.65 | 1.799 | 13.35 |
| 17 | 15.726 | 12.446 | 79.14 | 3.280 | 20.86 |
| 18 | 5.540 | 4.417 | 79.73 | 1.123 | 20.28 |
| 19 | 10.196 | 6.958 | 68.24 | 3.238 | 31.76 |
| 20 | 15.230 | 13.144 | 86.31 | 2.086 | 13.70 |

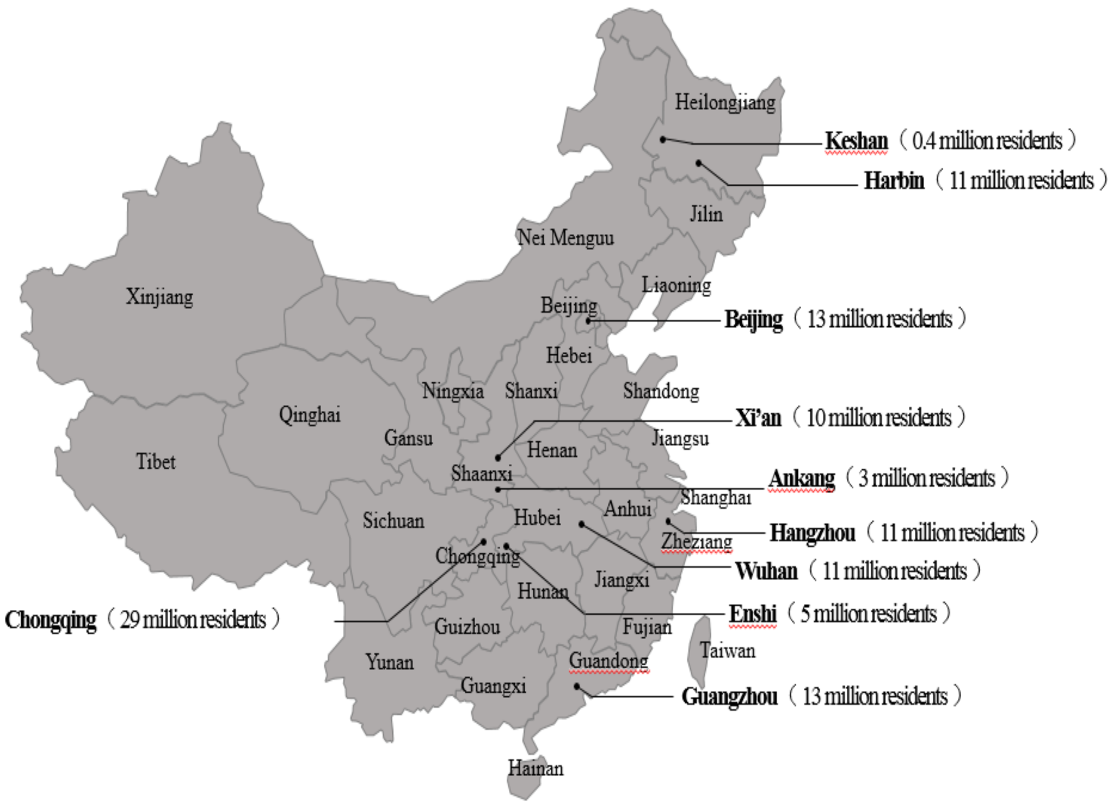


**Figure S1.** Map showing the geographical location and population of ten sampling areas in China where common rice and selenium-rich rice were collected. (Population data came from China's sixth census).
